# Supplementary material for: The Influence of Additives and Environment on Biodegradation of PHBV Biocomposites
Source: Polymers (Basel). 2022 Feb 21;14(4):838. doi: 10.3390/polym14040838 (PMC8963093; doi:10.3390/polym14040838)
Supplement: Supplementary file 1 [file polymers-14-00838-s001.zip › polymers-1577016-supplementary .pdf]

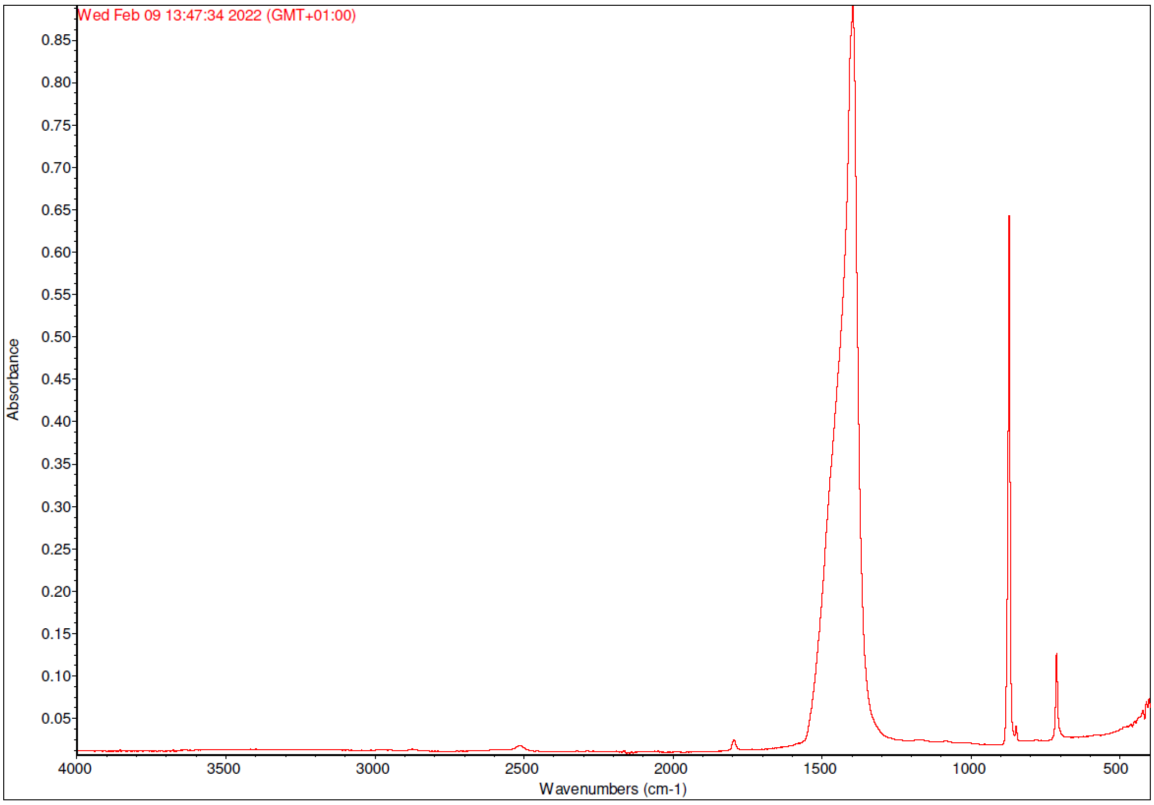

Figure S1. FTIR spectra of precipitated $CaCO_3$ (Honeywell Fluka, Seelze, Germany).

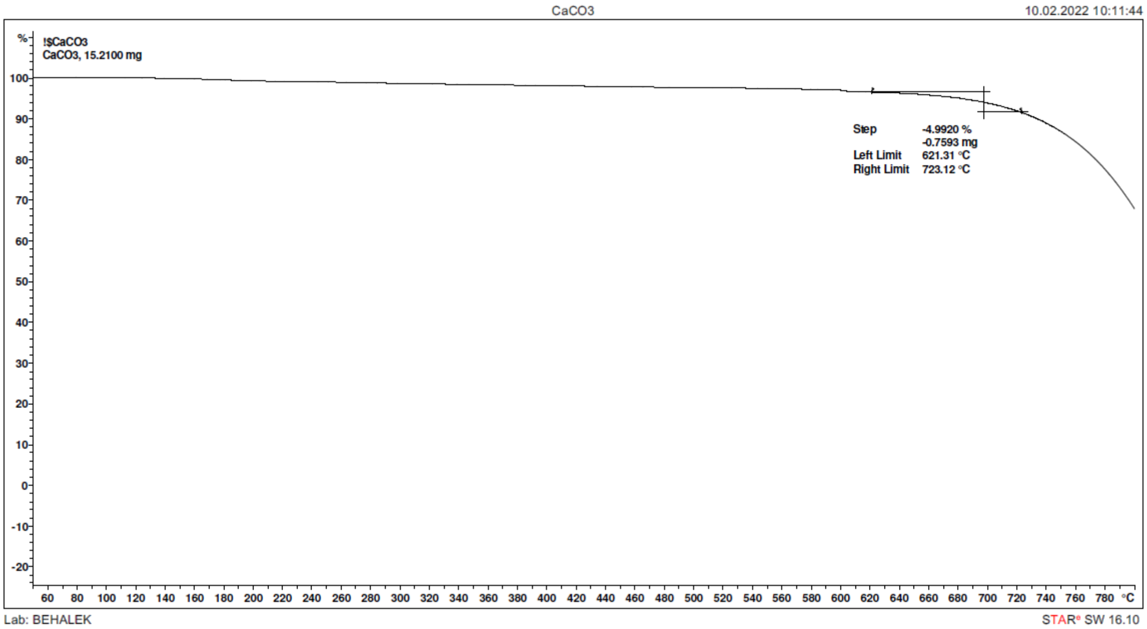

Figure S2. Thermogravimetric analysis (TGA) curve of precipitated $CaCO_3$ (Honeywell Fluka, Seelze, Germany).
